# High-throughput virtual screening, identification and *in vitro* biological evaluation of novel inhibitors of PLK1 and NRP1

**DOI:** 10.1080/14756366.2025.2514677

**Published:** 2025-08-18

**Authors:** Yang Xia, Yuting Chang, Lixia Guan, Yifei Geng, Qi Zhang, Xiaozhou Shen, Qian Bu, Miao-Miao Niu, Gaohua Han

**Affiliations:** ^a^ Department of Oncology, The Affiliated Taizhou People’s Hospital of Nanjing Medical University, Taizhou, China; ^b^ Department of Pharmaceutical Analysis, China Pharmaceutical University, Nanjing, China

**Keywords:** Polo-like kinase-1, neuropilin-1, lung cancer, dual-targeting inhibitors, virtual screening

## Abstract

Overexpression of PLK1 and NRP1 correlate with enhanced proliferative activity in lung cancer cells, thus the development of dual-target PLK1/NRP1 inhibitors holds great therapeutic promise. In this study, five compounds (PLN 1-5) targeting both PLK1 and NRP1 were identified using a multi-step virtual screening approach. PLN-5 showed nanomolar inhibitory potency against PLK1 (IC_50_ = 2.07 ± 0.13 nM) and NRP1 (IC_50_ = 5.15 ± 0.24 nM), exceeding the positive controls onvansertib and EG00229 by approximately 9-fold and 124-fold, respectively. Molecular dynamics (MD) simulations revealed that PLN-5 maintained a stable binding to the active sites of PLK1 and NRP1. Importantly, MTT assays showed that PLN-5 had significant antiproliferative activity (IC_50_ = 0.27 ± 0.02 μM) against human lung cancer cells, with no significant inhibitory effect on normal lung cells. In conclusion, these results demonstrate the therapeutic potential of PLN-5 as a dual-targeting antitumor agent that warrants further development.

## Introduction

Lung cancer (LC) is the second most common cancer with an annual incidence of 2 million worldwide and non-small cell lung cancer (NSCLC) accounts for up to 85% of all lung cancers^1–4^. There have been some advances in lung cancer treatment through surgery, chemotherapy and targeted therapy, and targeted therapy can significantly improve the prognosis of patients with genetic mutations^5^. However, despite improvements in these treatments, 5-year survival rates for patients with advanced NSCLC remain poor^6^. As single-target therapy is prone to drug resistance, dual-target drugs with high selectivity and low toxicity have become a promising strategy in cancer treatment.

**Figure 1.**
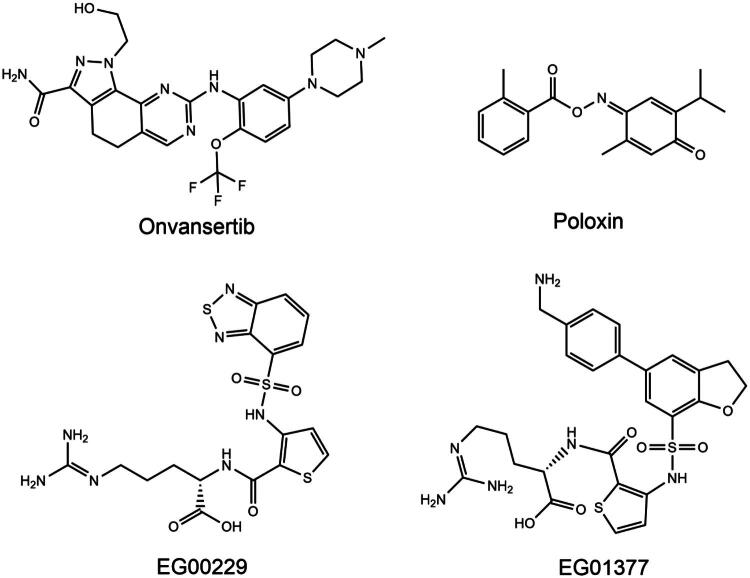
Reported PLK1 and NRP1 inhibitors.

Polo-like kinase-1 (PLK1) is a conserved serine/threonine kinase that regulates mitosis and cytokinesis^7^^,^^8^. Its structure consists of an N-terminal catalytic kinase domain and a C-terminal polo-box domain (PBD)^9^. PLK1 plays an important role in the precise maintenance of genome stability in response to mitosis, spindle assembly and DNA damage^10^^,^^11^. Sufficient evidence suggests that PLK1 mediates the phosphorylation process of dozens of proteins such as cyclin B, Cdc25C, TCTP and BubR1^12^. PLK1 has been found to be highly expressed in a variety of human malignancies including NSCLC, and a close relationship between PLK1 and tumorigenesis has been demonstrated^13–15^. ATP competitive small molecule inhibitors typically block kinase activity^16^. Several PLK1 ATP competitive inhibitors have been identified, such as onvansertib (Figure 1)^17^^,^^18^. In addition, targeting inhibition of the polo-box structural domain (PBD) of PLK1 proved to be a more versatile approach, which resulted in mislocalization of PLK1 in cells^19–21^. Targeting PLK1 PBD also avoids the selectivity issues of ATP-competitive inhibitors^22^^,^^23^. For example, poloxin (Figure 1), thymoquinone are existing non-ATP competitive PLK1 inhibitors^21^^,^^24^.

Neuropilin 1 (NRP1) is a highly conserved multifunctional type I single-pass transmembrane protein^25^. NRP1 binds to various extracellular ligands, including vascular endothelial growth factor (VEGF) and transforming growth factor β1, leading to tumour angiogenesis and cell migration^26–28^. NRP1 is also expressed by a variety of immune cells and is actively involved in immune functions^29^. It has been shown that NRP1 is a key co-receptor for VEGF-mediated NSCLC cell survival and tumour growth^30^. Moreover, NRP1 was overexpressed in metastatic NSCLC tissues^31^^,^^32^. Inhibition of NPR1 blocks TGF-β1-induced epithelial-mesenchymal transition (EMT) in NSCLC cells^31^. A number of peptide antagonists of NRP1 are known, such as the small molecule drugs EG00229 and EG01377 (Figure 1), which showed good activity in the lower micromolar range^33^^,^^34^.

PLK1 has been found to be involved in the mechanism of resistance to multiple chemotherapeutic agents such as paclitaxel^35–38^. Therefore, attempts to simultaneously target PLK1 as well as other pathways important for specific cancers may be effective in improving the anticancer efficacy of drugs^39^. Both PLK1 and NRP1 are significantly overexpressed in NSCLC^40^^,^^41^. Research has shown that PLK1 promotes NSCLC metastasis by enhancing the TGF-β signalling pathway and is a key driver of EMT in NSCLC^42^. As a key regulator of TGF-β signalling, inhibition of NRP1 effectively blocks TGF-β1-induced EMT^31^. These findings suggest that PLK1 and NRP1 may cooperatively promote the invasive and metastatic capabilities of lung cancer cells through synergistic regulation of EMT-related transcription factor expression and activity. Considering that PLK1 and NRP1 are essential for lung cancer development, targeting PLK1 and NRP1 simultaneously represents a potential therapeutic approach for lung cancer. However, drug combination therapies are typically accompanied by drug-drug interactions and side effects^43^^,^^44^. Compared to combination therapies, single molecule dual inhibitors can achieve comparable efficacy with reduced risk profiles^45^. Thus, we aimed to develop dual-target PLK1/NRP1 inhibitors as antitumor agents. Currently, there are no reported inhibitors that dual-target PLK1 and NRP1.

Structure-based virtual screening is a novel approach to identifying new scaffolds for specific binding sites of known target protein structures^46^. Molecular docking allows the prediction of ligand conformations within protein binding pockets, and the energy changes that occur in intermolecular interactions can be quantitatively predicted^47^. Pharmacophore screening is based on the characteristics of the ligand to screen for compounds that are active against the same target^48^. The combined screening of molecular docking and pharmacophore modelling could be effective in discovering lead compounds. In previous studies, we successfully identified a highly potent inhibitor of tubulin/PARP-1 and a cyclic peptide inhibitor of NRP1/KRAS^G12D^ using structure-based virtual screening^49^^,^^50^. Here, we identified novel dual-targeted PLK1/NRP1 inhibitors (PLN 1–5) using an integrated screening approach. Enzyme inhibition assays indicated that PLN 1-5 inhibited both PLK1 and NRP1 in the nanomolar range. The binding stability of PLN-5 within the active domains of PLK1 and NRP1 was verified by MD simulations. In addition, PLN-5 exhibited excellent *in vitro* antiproliferative activity. Thus, dual-target PLK1/NRP1 inhibitor (PLN-5) may represent a potential therapeutic strategy in lung cancer.

## Materials and methods

### Cell culture and materials

The lung cancer cells NCI-H460 (large cell lung carcinoma), A549 (adenocarcinoma), A427 (adenocarcinoma) and normal lung cells BEAS-2B (human normal lung epithelial cells) were purchased from The American Type Culture Collection (ATCC) (Manassas, VA, USA). The cells were cultured with Roswell Park Memorial Institute (RPMI) ‐1640 medium. The temperature of the cell culture was maintained at 37 °C. Hit compounds were obtained commercially from WuXi AppTec (PLN-1, Lot NO: P112561-P1; PLN-2, Lot NO: P112561-P2; PLN-3, Lot NO: P112561-P3; PLN-4, Lot NO: P112561-P4; PLN-5, Lot NO: P112561-P5) (Table S1). The purity of PLN 1-5 was more than 98% as determined by HPLC (Supplementary material). Human PLK1 and NRP1 proteins were purchased from Abcam (Cambridge, MA, USA).

### Pharmacophore construction

The ligand-bound structures of PLK1 (PDB ID: 3THB) and NRP1 (PDB ID: 3I97) were downloaded from the Protein Data Bank (PDB) and analysed by molecular modelling using the Molecular Operating Environment (MOE). First, the structures were optimised using QuickPrep module of MOE. Subsequently, protein-ligand interaction patterns were analysed using the Ligand Interaction tool in MOE. In view of the above analysis, pharmacophore models were constructed using Pharmacophore Query Editor of the MOE.

### Virtual screening

A large compound database containing 116,490 small molecules was generated using combinatorial chemistry approaches. All 2D molecular structures were then converted into energetically optimised 3D conformations using the energy minimisation tool of MOE. The compound database was initially filtered through the established PLK1 pharmacophore model. Subsequently, molecular docking was performed using MOE’s Dock module to assess the binding affinity of the pharmacophore-filtered compounds against both the PLK1 and NRP1 active sites. The docking was determined by: (i) ligand-receptor pose generation using the Triangle Matcher method, and (ii) binding energy estimation using the London dG scoring function. Lower docking scores are indicative of a higher binding affinity.

### PLK1 inhibition assay

The method was as previously described^51^. Briefly, the enzyme reaction mixture containing different doses of compounds was incubated for 90 min at room temperature. Then the mixture was transferred to a Neutravidin-coated 384-well plate and incubated for 60 min at room temperature. Then the wells were incubated with 50 μL of the antibody mixture for 1 h. The bound europium was liberated using 50 μL of Enhancement Solution.

### NRP1 inhibition assay

The method was as previously described^33^. Briefly, HUVEC cells transfected with NRP1 were added to a 96-well plate and after growing to confluence, and the compounds were added to each well at different concentrations. Then, the ^125^I-VEGF-A165 (0.1 nM) was added to measure non-specific binding. Data curves were plotted and half maximal inhibitory concentrations (IC_50_) were derived for the selected compounds using Prism 6.0 software.

### In vitro selectivity assay

To assess the selectivity of PLN-5, we systematically determined its inhibitory activity against PLK2, PLK3, PLK4 (homologues of PLK1) and NPR2 (homologue of NPR1). This assay was performed by ICE Bioscience Inc (Beijing, China).

### MD simulations

The structural models of PLN-5 bound to PLK1 and NRP1 were constructed using MOE, respectively. MD simulations of the PLK1-PLN-5 and NRP1-PLN-5 complexes were performed using GROMACS. The topology file of PLN-5 was generated by the Acpype server (www.bio2byte.be). The system was dissolved in a 1.0 nm cubic box by the SPC/E water model. Energy minimisation was performed by steepest descent (5000 steps). In a 100 ps NVT simulation, the system temperature was maintained at 300 K using a V-rescale thermostat. In a 100 ps NPT simulation, a Parinello-Rahman barostat was further conducted to keep the system pressure at 1 bar. Finally, 50 ns MD simulations were performed, and the data obtained were processed through Prism 6.0 software.

### MTT assay

Cells were seeded in 96-well plates (5 × 10^4^ cells/well) and incubated overnight. Cells were incubated with different concentrations of PLN-5 at 37 °C for 72 h. After removal of the medium, MTT stock solution (5 mg/ml) was added and the incubation continued for 4 h. After centrifugation, the insoluble crystals were dissolved in dimethyl sulfoxide and gently shaken for 15 min. Finally, absorbance was measured at 570 nm using a microplate reader. The IC_50_ were calculated using Prism 6.0 software.

## Results and discussion

### PLK1 pharmacophore model construction and validation

**Figure 2.**
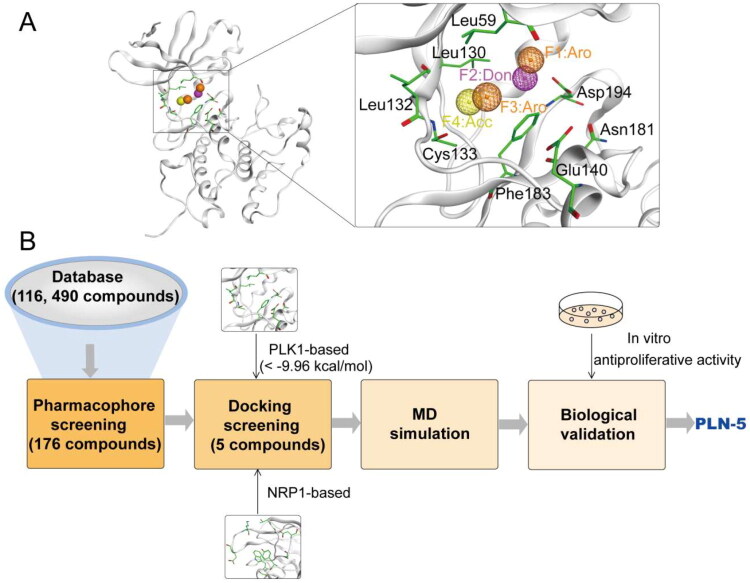
(A) Pharmacophore models of PLK1. Residues in the binding site of PLK1 are shown as green sticks. (B) The workflow of multi-step virtual screening.

Pharmacophore modelling identifies key features for molecular recognition. The pharmacophore model was constructed based on the structure of PLK1 (PDB ID: 3THB) in complex with its ligand. As depicted in Figure 2(A), the two Aro features corresponded to hydrophobic interactions formed with residues Leu59, Leu130, Leu132, and Phe183. The Don and Acc features correspond to hydrogen bonding interactions formed by the oxygen atom of Asp194 and the nitrogen atom of Cys133, respectively. These interactions highlight the critical role of hydrophobic and hydrogen bonding in protein-ligand binding. Finally, four pharmacophore features were constructed, including two aromatic centre features (F1 and F3: Aro, orange color), a hydrogen bond donor feature (F2: Don, purple color) and a hydrogen bond acceptor feature (F4: Acc, yellow color).

To validate the reliability of the constructed pharmacophore model, we evaluated the model using a test set database containing 1000 molecules, including 13 known active PLK1 inhibitors^51^^,^^52^. The pharmacophore model was employed as a three-dimensional search template for virtual screening, and key validation parameters such as the Güner-Henry (GH) score were calculated. As shown in Table 1, the GH score of the model was 0.9 (typically with a threshold >0.7 indicating good pharmacophore model performance), confirming that the pharmacophore model can effectively discriminate between active and inactive molecules.

**Table 1.** Validation of pharmacophore model using the GH score method.

Parameter	Pharmacophore Model
Total molecules in database (D)	1000
Total number of actives in database (A)	13
Total hits (Ht)	15
actives hits (Ha)	13
% Yield of actives ((Ha/Ht) × 100)	87%
% Ratio of actives ((Ha/A) × 100)	100%
Enrichment factor (E) ((Ha × D)/(Ht × A))	67
False negatives (A − Ha)	0
False positives (Ht-Ha)	2
Goodness of hit score (GH)	0.90

### Virtual screening

**Figure 3.**
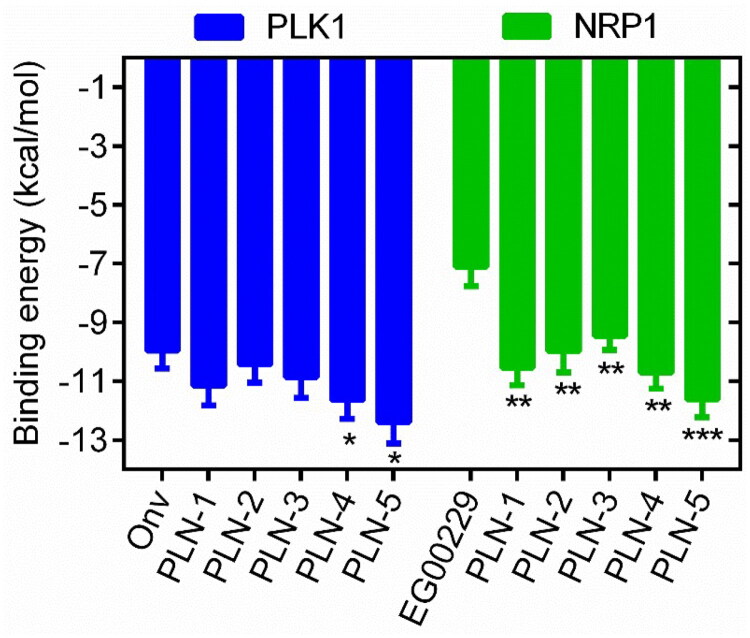
The binding free energy (kcal/mol) of PLN 1-5 with PLK1 and NRP1, respectively. Onvansertib and EG00229 were used as positive controls. For PLK1 group, *p < 0.05 means a significant difference versus Onv. For NRP1 group, **p < 0.01, ***p < 0.001 means a significant difference versus EG00229.

**Figure 4.**
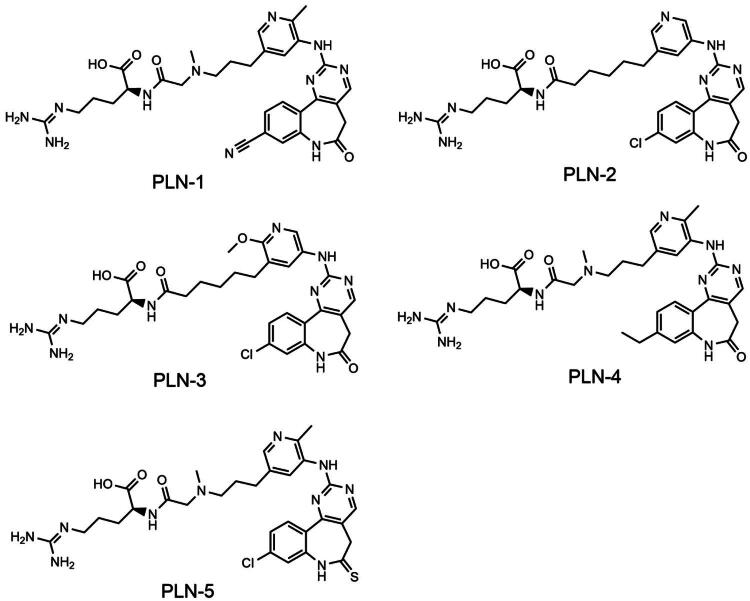
The chemical structures of PLN 1-5.

The virtual screening flowchart is shown in Figure 2(B). Dual PLK1/NRP1 inhibitors were screened from a database containing 116, 490 compounds. Based on the established pharmacophore features, we performed database screening and identified 176 compounds with satisfactory pharmacophore match. Then, these 176 compounds were screened for molecular docking based on the structural domain of PLK1. The binding affinities of the compounds to PLK1 were assessed using docking scores, with lower scores correlating with stronger binding interactions. A docking score of −9.96 kcal/mol for onvansertib was used as the cut-off value. Therefore, we selected 34 compounds with binding free energies below −9.96 kcal/mol. Subsequently, the 34 hits were further docked to NRP1 to assess their binding potential. Finally, the top 5 compounds (PLN 1-5) with the lowest scores for NRP1 docking were selected (Figure 3). PLN 1-5 showed a lower binding free energy than the positive control, indicating a strong binding affinity. The structures of PLN 1-5 are shown in Figure 4.

**Figure 5.**
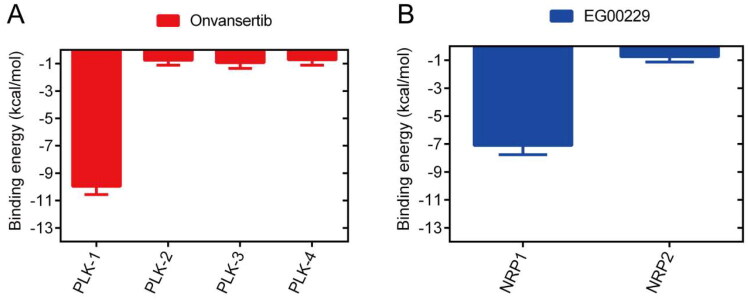
The binding free energy (kcal/mol) of onvansertib with PLKs 1–4 and EG00229 with NRPs 1-2.

To validate the selectivity of the molecular docking screening approach for PLK1 and NRP1, we performed docking simulations using the known selective PLK1 inhibitors onvansertib^53^ and NRP1 inhibitors EG00229^33^. As shown in Figure 5, comparative binding free energy analysis revealed significantly higher values for onvansertib against PLK2, PLK3 and PLK4, while EG00229 showed an elevated binding free energy towards NRP2, indicating a low binding affinity. These results demonstrate the selectivity profile of the molecular docking screening methodology.

### Interaction analysis

**Figure 6.**
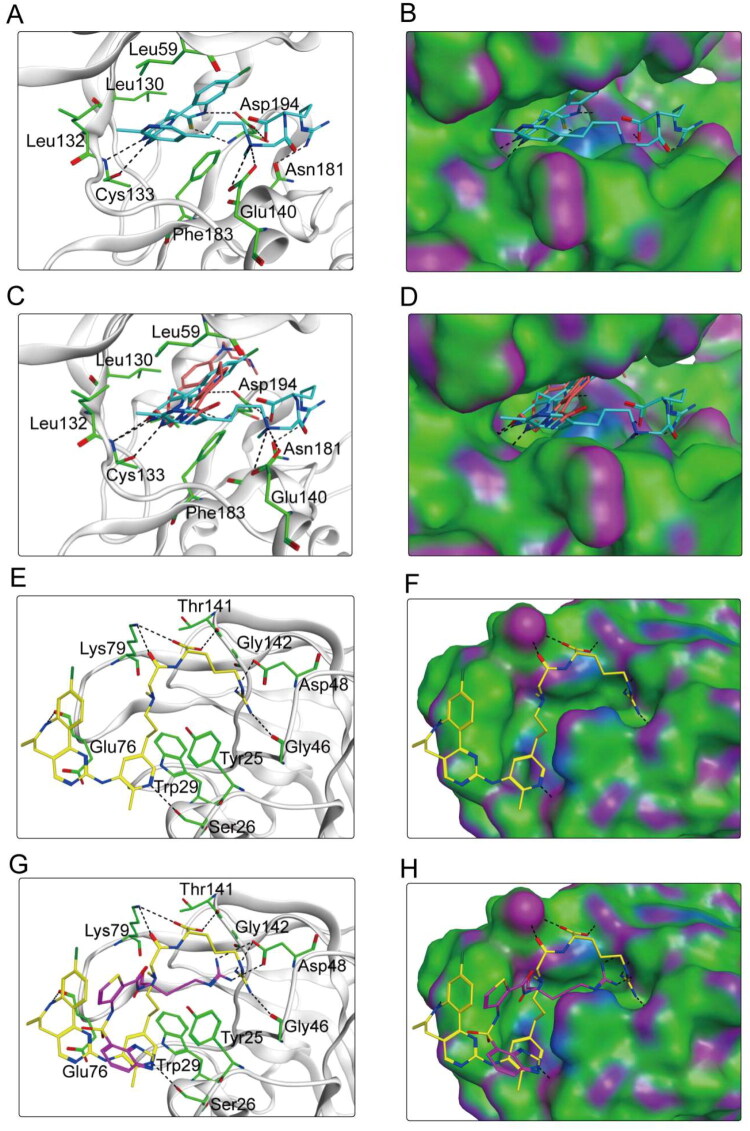
(A,B) The binding mode of PLN-5 (cyan sticks) within the binding site of PLK1. (C,D) The binding mode of onvansertib (orange sticks) within the binding site of PLK1. (E,F) The binding mode of PLN-5 (yellow sticks) within the binding site of NRP1. (G,H) The binding mode of EG00229 (purple sticks) within the binding site of NRP1. Binding site residues are displayed in green sticks. The hydrogen bonds are depicted with black dashed lines.

Since PLN-5 had the strongest binding affinity with PLK1 and NRP1 in molecular docking, the detailed binding modes with the target protein were analysed. The binding mode of PLN-5 docked to PLK1 was shown in Figure 6(A,B). PLN-5 formed eight hydrogen bonds with Cys133, Asn181, Glu140, and Asp194, which determined the orientation of the binding process. Meanwhile, PLN-5 created hydrophobic interactions with the amino acids Leu59, Leu130, Leu132 and Phe183, stabilising it in the hydrophobic pocket of PLK1. Figure 6(C,D) shows that the positive control onvansertib formed only one hydrogen bond with PLK1. This binding pattern suggests that PLN-5 has enhanced binding stability to PLK1 compared to onvansertib. Figure 6(E,F) shows the binding mode of PLN-5 docked to NRP1 protein. PLN-5 formed seven hydrogen bonds with Ser26, Gly46, Asp48, Glu76, Lys79, and Thr141, contributing to the stabilisation of PLN-5 at the NRP1 binding site. PLN-5 created critical hydrophobic interactions with the residues Tyr25, Trp29 and Gly142 of NRP1 to increase the binding affinity. Meanwhile, PLN-5 showed excellent complementarity with the pocket of NRP1. As shown in Figure 6(G,H), the positive control EG00229 formed two hydrogen bonds with NRP1, a significantly lower number than that exhibited by PLN-5. In conclusion, the results suggest that PLN-5 can stably bind to the active pockets of both PLK1 and NRP1.

### Inhibitory effects on PLK1 and NRP1

The inhibitory activity of PLN 1-5 against PLK1 and NRP1 was assessed by enzyme inhibition assays using the PLK1 inhibitor onvansertib and the NRP1 inhibitor EG00229 as positive controls. As illustrated in Table 2, PLN 1-5 exhibited dual-target inhibitory potency with nanomolar inhibition of both PLK1 and NRP1, and the IC_50_ values of PLN 1-5 were lower than the positive control. Among them, PLN-5 had the strongest inhibitory effect, and the IC_50_ values for PLK1 (IC_50_ = 2.07 ± 0.13 nM) and NRP1 (IC_50_ = 5.15 ± 0.24 nM) were approximately 9-fold higher than that of onvansertib and 124-fold higher than that of EG00229. To assess the selectivity profile of PLN-5, we systematically evaluated its inhibitory activity against PLK2, PLK3, PLK4 and NPR2. As shown in Table 3, PLN-5 showed almost no inhibitory activity (IC_50_ > 10 μM) against PLK2, PLK3, PLK4 and NRP2, confirming its good target selectivity. These results indicate that PLN-5 has potent inhibitory activity against PLK1 and NRP1 with excellent target specificity. Meanwhile, the inhibitory results are consistent with molecular docking studies showing that PLN-5 is the most promising compound to inhibit PLK1 and NRP1.

**Table 2.** Dual inhibitory effects of PLN 1–5 on PLK1 and NRP1.

Compounds	PLK1 (IC_50_, nM)	NRP1 (IC_50_, nM)
PLN-1	8.12 ± 0.34	12.69 ± 0.81
PLN-2	16.53 ± 2.86	24.27 ± 1.29
PLN-3	11.38 ± 0.74	30.48 ± 2.55
PLN-4	4.51 ± 0.26	7.83 ± 0.42
PLN-5	2.07 ± 0.13	5.15 ± 0.24
Onvansertib	19.54 ± 2.32	no inhibition
EG00229	no inhibition	639.71 ± 20.84

The data are presented as the mean ± SD, *n* = 3.

**Table 3.** Inhibitory effects of PLN-5 on PLK2, PLK3, PLK4 and NRP2.

Name	IC_50_ (μM)			
	PLK2	PLK3	PLK4	NRP2
PLN-5	>10	>10	>10	>10

The data are presented as the mean ± SD, *n* = 3.

### MD simulation

**Figure 7.**
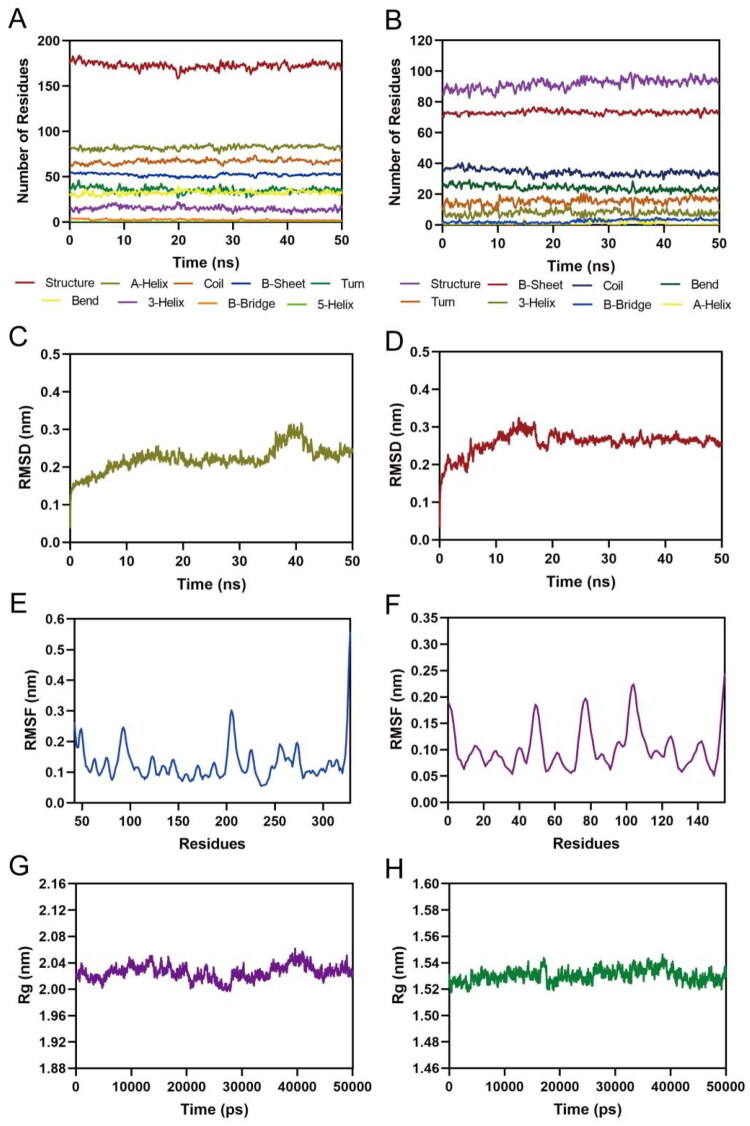
MD simulation of PLN-5 in complex with PLK1 and NRP1. (A,B) The secondary structures analysis of PLK1 and NRP1. (C,D) The RMSD of the PLK1-PLN-5 complex and NRP1-PLN-5 complex. (E,F) The RMSF of the Cα atoms of PLK1 and NRP1. (G,H) Radius of Gyration of PLK1 and NRP1.

The binding stability of the PLK1-PLN-5 and NRP1-PLN-5 complexes was further investigated by MD simulation. Figure 7(A,B) showed that the secondary structures of the PLK1 and NRP1 remained stable throughout the simulation, indicating that PLN-5 binding does not affect protein stability. The Root Mean Square Deviation (RMSD) is related to the stability of the complex. Figure 7(C,D) illustrated the RMSD of the PLK1-PLN-5 and NRP1-PLN-5 complexes for the atoms in a 50 ns MD simulation, respectively. The RMSD of PLK1-PLN-5 stabilised at around 0.2 nm after 10 ns, and that of NRP1-PLN-5 stabilised at around 0.27 nm after 20 ns. The results show that the binding between PLN-5 and PLK1/NRP1 is stable. The Root Mean Square Fluctuation (RMSF) indicates the flexibility and strength of the protein amino acid movement. The lower the RMSF value, the less movement is produced by the amino acid residues. As seen in Figure 7(E,F), the RMSF of PLK1 for the key residues (Leu59, Leu130, Leu132, Cys133, Glu140, Asn181, Phe183 and Asp194) were all less than 0.13 nm, and the RMSF of NRP1 for the key residue (Ser26, Gly46, Asp48, Glu76, Lys79 and Thr141) were all less than 0.25 nm. These results demonstrate that key residues of PLK1 and NRP1 bind stably to PLN-5. Additionally, Figure 7(G,H) showed that the variation in the radius of gyration (Rg) values for both PLK1 and NRP1 was less than 0.04 nm, suggesting that the proteins remained structurally compact throughout the simulation. Therefore, MD results show that PLN-5 could interact with the active sites of PLK1 and NRP1 with high binding stability.

### In vitro cellular assays

**Figure 8.**
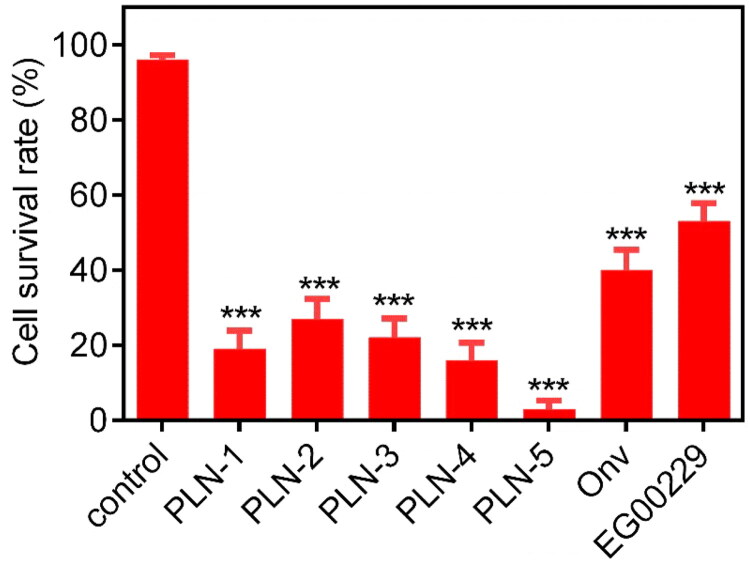
The cell survival rate (%) of PLN 1–5 on NCI-H460 cells. Onvansertib and EG00229 are used as positive control. NCI-H460 cells were treated with 2 μM of PLN 1–5, onvansertib and EG00229. Results are expressed as mean ± SD, *n* = 3. ***p < 0.001 means a significant difference versus the control group.

The antiproliferative effects of PLN-5 on lung cancer cells and normal lung cells were assessed using the MTT assay. The experimental data are presented in Table 4. PLN-5 exhibited significant antiproliferative activity in all lung cancer cell lines (IC_50_ = 0.27 ± 0.02 μM for NCI-H460, IC_50_ = 0.41 ± 0.03 μM for A549, and IC_50_ = 0.33 ± 0.02 μM for A427). In addition, the inhibitory activity of PLN-5 on human lung cancer cells was significantly higher than that on human normal lung cells BEAS-2B (IC_50_ > 10 μM). Subsequently, NCI-H460 cells were treated with 2 μM of PLN 1-5, onvansertib and EG00229, respectively. The survival rate of NCI-H460 cells treated with PLN 1-5 was all lower than that of the control drugs (Figure 8). In particular, the NCI-H460 cells treated with PLN-5 showed the lowest survival rate. This evidence suggests that PLN-5 has anticancer potency *in vitro* with no apparent toxicity to normal lung cells.

**Table 4.** The cytotoxicity of PLN-5 on lung cancer cells and human normal lung cells.

Name	IC_50_ (μM)^a^			
	NCI-H460	A549	A427	BEAS-2B
PLN-5	0.27 ± 0.02	0.41 ± 0.03	0.33 ± 0.02	>10

^a^ IC_50_ (μM) is the concentration of compound needed to reduce cell growth by 50% following 72 h cell treatment with PLN-5. The data are presented as the mean ± SD, *n* = 3.

## Conclusions

Given the pivotal role of PLK1 and NRP1 in promoting lung cancer proliferation, dual inhibition of PLK1 and NRP1 may serve as a potential therapeutic strategy for lung cancer. Compared to single-target or combination therapies, dual-target drugs with a single chemical entity have more predictable pharmacokinetics (PK) and pharmacodynamics (PD). Therefore, dual-targeted therapies with high safety and efficacy significantly reduce patient resistance and toxicities. In this study, the first dual-target PLK1/NRP1 inhibitors were successfully screened through an integrated virtual screening protocol. PLN-5 was successfully identified as a highly potent inhibitor, which showed significant inhibitory activity against both PLK1 and NRP1. MD simulation proved that PLN-5 could stably bind to both PLK1 and NRP1. Moreover, MTT assays confirmed that PLN-5 exhibited the most superior antiproliferative activity, with an inhibition rate of more than 90% against NCI-H460 cells. The experimental results show that the integrated virtual screening approach significantly improved lead discovery efficiency. Given the significant antiproliferative activity of PLN-5, there is a need to further evaluate its therapeutic potential by establishing a xenograft tumour model system to assess its *in vivo* antitumor efficacy and safety. In conclusion, PLN-5 is a potent dual-target inhibitor of PLK1 and NRP1 and is expected to be further developed for the treatment of lung cancer.

## Supplementary Material

Supplementary Material for review.docx

## Data Availability

The data presented in the current study are available from the corresponding author upon reasonable request.
