# Supplementary material for: High-throughput virtual screening, identification and in vitro biological evaluation of novel inhibitors of PLK1 and NRP1
Source: J Enzyme Inhib Med Chem. 2025 Aug 18;40(1):2514677. doi: 10.1080/14756366.2025.2514677 (PMC12364115; doi:10.1080/14756366.2025.2514677)
Supplement: Supplementary Material for review.docx [file IENZ_A_2514677_SM2587.docx]

**Supporting Information**

**High-throughput virtual screening, identification and in vitro biological evaluation of novel inhibitors of PLK1 and NRP1**

**Table S1.** Lot numbers of the identified compounds.

| **Compounds** | **Lot NO** |
| --- | --- |
| PLN-1 | P112561-P1 |
| PLN-2 | P112561-P2 |
| PLN-3 | P112561-P3 |
| PLN-4 | P112561-P4 |
| PLN-5 | P112561-P5 |

**HPLC chromatograms of PLN 1-5**

**HPLC chromatogram of PLN-1**

| Peak No. | Ret Time | Area | Conc. |
| --- | --- | --- | --- |
| 1 | 8.805 | 9099.896 | 0.0845 |
| 2 | 9.220 | 41387.672 | 0.3843 |
| 3 | 9.445 | 10567243.000 | 98.1332 |
| 4 | 9.445 | 50501.895 | 0.4690 |
| 5 | 11.947 | 100035.352 | 0.9290 |
| Total |  |  | 100.00 |

**HPLC chromatogram of PLN-2**

| Peak No. | Ret Time | Area | Conc. |
| --- | --- | --- | --- |
| 1 | 7.997 | 12702.511 | 0.3258 |
| 2 | 8.387 | 6950.449 | 0.1783 |
| 3 | 8.602 | 3723.697 | 0.0955 |
| 4 | 8.932 | 8626.360 | 0.2213 |
| 5 | 9.205 | 14391.334 | 0.3691 |
| 6 | 9.675 | 5114.005 | 0.1312 |
| 7 | 9.913 | 3847368.750 | 98.6789 |
| Total |  |  | 100.00 |

**HPLC chromatogram of PLN-3**

| Peak No. | Ret Time | Area | Conc. |
| --- | --- | --- | --- |
| 1 | 6.613 | 473.148 | 0.0150 |
| 2 | 9.367 | 9278.144 | 0.2947 |
| 3 | 9.975 | 2157.199 | 0.0685 |
| 4 | 10.130 | 939.079 | 0.0298 |
| 5 | 10.292 | 3095701.750 | 98.3188 |
| 6 | 10.737 | 14458.613 | 0.4592 |
| 7 | 11.285 | 1303.700 | 0.0414 |
| 8 | 17.113 | 4541.807 | 0.1442 |
| 9 | 17.492 | 7914.109 | 0.2514 |
| 10 | 20.182 | 11868.195 | 0.3769 |
| Total |  |  | 100.00 |

**HPLC chromatogram of PLN-4**

| Peak No. | Ret Time | Area | Conc. |
| --- | --- | --- | --- |
| 1 | 4.693 | 12486.685 | 0.2239 |
| 2 | 8.928 | 6441.975 | 0.1155 |
| 3 | 9.238 | 28358.355 | 0.5086 |
| 4 | 9.465 | 5482062.500 | 98.3102 |
| 5 | 9.465 | 38586.672 | 0.6920 |
| 6 | 12.138 | 8357.400 | 0.1499 |
| Total |  |  | 100.00 |

**HPLC chromatogram of PLN-5**

| Peak No. | Ret Time | Area | Conc. |
| --- | --- | --- | --- |
| 1 | 9.480 | 4614863 | 98.12 |
| 2 | 10.218 | 34016 | 0.7233 |
| 3 | 11.003 | 54233 | 1.153 |
| Total |  |  | 100.00 |
